# Generalist camouflage can be more successful than microhabitat specialisation in natural environments

**DOI:** 10.1186/s12862-021-01883-w

**Published:** 2021-08-03

**Authors:** Emmanuelle Sophie Briolat, Lina María Arenas, Anna E. Hughes, Eric Liggins, Martin Stevens

**Affiliations:** 1 Centre for Ecology and Conservation, University of Exeter, Penryn Campus, TR10 9FE Penryn, UK; 2 Department of Psychology, University of Essex, Wivenhoe House, CO4 3SQ Colchester, UK; 3 QinetiQ, Cody Technology Park, Ively Road, Farnborough, GU14 0LX Hampshire UK

**Keywords:** Camouflage, Crypsis, Background matching, Anti-predator coloration, Detection risk, Citizen science

## Abstract

**Background:**

Crypsis by background-matching is a critical form of anti-predator defence for animals exposed to visual predators, but achieving effective camouflage in patchy and variable natural environments is not straightforward. To cope with heterogeneous backgrounds, animals could either specialise on particular microhabitat patches, appearing cryptic in some areas but mismatching others, or adopt a compromise strategy, providing partial matching across different patch types. Existing studies have tested the effectiveness of compromise strategies in only a limited set of circumstances, primarily with small targets varying in pattern, and usually in screen-based tasks. Here, we measured the detection risk associated with different background-matching strategies for relatively large targets, with human observers searching for them in natural scenes, and focusing on colour. Model prey were designed to either ‘specialise’ on the colour of common microhabitat patches, or ‘generalise’ by matching the average colour of the whole visual scenes.

**Results:**

In both the field and an equivalent online computer-based search task, targets adopting the generalist strategy were more successful in evading detection than those matching microhabitat patches. This advantage occurred because, across all possible locations in these experiments, targets were typically viewed against a patchwork of different microhabitat areas; the putatively generalist targets were thus more similar on average to their various immediate surroundings than were the specialists.

**Conclusions:**

Demonstrating close agreement between the results of field and online search experiments provides useful validation of online citizen science methods commonly used to test principles of camouflage, at least for human observers. In finding a survival benefit to matching the average colour of the visual scenes in our chosen environment, our results highlight the importance of relative scales in determining optimal camouflage strategies, and suggest how compromise coloration can succeed in nature.

**Supplementary Information:**

The online version contains supplementary material available at 10.1186/s12862-021-01883-w.

## Background

Predation is a major selective pressure shaping the visual appearance of animals, and many species use some form of protective coloration to enhance their survival [1, 2]. Colour patterns can function at several stages in an encounter with predators, from preventing detection and recognition to reducing the likelihood of successful capture [3], but most undefended animals rely on camouflage to avoid initiation of an attack [4, 5]. While a number of strategies have been proposed and shown to reduce the risk of attack, such as disruptive coloration [6], countershading [7], and transparency [8], perhaps the most obvious way to achieve crypsis is background-matching camouflage, whereby prey animals closely resemble the visual characteristics of the background against which they are seen [3, 9]. Long-recognised and discussed by key thinkers in the field, from Erasmus Darwin to Abbott Thayer [5, 10], background-matching is widespread in nature, and has been shown to reduce detection by predators and promote survival in the wild (e.g. [11–13]). Yet, although the principle of background-matching seems simple, defining the background which prey should match is not. Animals generally inhabit heterogenous natural environments, which often also change seasonally, and some species must move between visually-distinct habitat patches to find food or mates; maintaining a good resemblance to all these potential backgrounds is challenging [9, 14, 15]. Behavioural adaptations, such as adjusting pattern orientation [16] or altering the background itself [17], can improve background-matching against heterogeneous natural backgrounds [18], while some species can even change their appearance to better adapt to their current environment [19]. However, for species with fixed colour patterns, how camouflage should be optimised in heterogeneous habitats remains an evolutionary puzzle.

Broadly, prey in variable environments can either specialise on one visual background type, at the expense of appearing more visible against others, or utilise a generalist strategy, a ‘compromise’ colour pattern that matches several possible backgrounds somewhat but none as strongly as specialists might achieve [14, 15]. Theoretical models considering an environment with two distinct microhabitat patches predict that both strategies can be advantageous, depending on the distribution of patches, prey and predators, and especially on the shape of the trade-off between concealment in one microhabitat versus the other [14, 20]. This relationship is determined by the visual capabilities of predators, constraints in prey patterning and background properties such as visual similarity between microhabitats: generalists are more likely to be successful across microhabitats that are sufficiently similar for some level of crypsis to be possible in both, while specialists may fare better if patches are more divergent. This prediction is generally borne out by empirical tests [21, 22], but the specific nature of differences between background patches can affect the outcome. In a series of computer-based experiments with humans, in which target patterns evolved according to an artificial evolution algorithm dependent on participant reaction times, generalists were favoured when targets were seen against backgrounds differing in pattern size, but specialists did better when backgrounds varied in luminance [23], suggesting that humans may be more sensitive to mismatches in colour and luminance than pattern [15]. Other background characteristics are also important in determining the optimal solution, such as the relative scale of the target and background microhabitat patches [5]: experiments with avian predators found generalists were more successful on backgrounds with fine-grained patterns, while specialists fared better on more coarsely-patterned backgrounds [24].

Observations of real animal patterns suggest that a range of strategies exist between the two extremes, reflecting variation in prey and predator traits, and the visual environment. An abundance of studies demonstrate a close match between the colour patterns of animals and their chosen backgrounds at a range of spatial scales (e.g. in moths [13], rodents [25, 26], lizards [27], and even plants [28]). Yet there is also variation in how closely different species or populations match their immediate backgrounds, and often local individual variation in form, suggesting a continuum of strategies from specialist to generalist, as seen among North American moth species [29], or across populations of wall lizards (*Podarcis erhardii*), which show a lower degree of specialisation on volcanic islands with historically more variable backgrounds [27]. Some mammals, such as *Peromyscus* mice, show strong local adaptation in pelage colour, providing protection against visually-guided predators [11, 30, 31], but the widespread dull brown and grey coloration of most mammals [32, 33] intuitively seems to be suited to camouflage across many habitats, at least to human observers.

Conclusively demonstrating that natural colour patterns function as compromise camouflage is challenging [5], but recent studies suggest that some species occupying several visually distinct habitats adopt generalist patterns, matching a whole set of potential backgrounds, or a mix of features enabling a degree of both local adaptation and global matching. For example, pelage colour in desert rodents such as gerbils (*Gerbillus* spp.) and African desert jerboas (*Jaculus jaculus*) has been shown to correlate with large-scale measures of habitat coloration across their range [26, 34], but recent work shows that these animals are better matched in colour and luminance to a global set of potential backgrounds within their range than to the specific ones they were found on [35]. Despite some variety among desert habitats, rodent pelage has relatively low chromatic contrasts against all of them, for both avian and mammalian predator visual systems, so matching a global set of backgrounds should enable a good level of crypsis in all situations. Similarly, in shore crabs (*Carcinus maenas*) found on diverse coastal habitats, from mud flats to rock pools, the colour and pattern of juveniles correlates with their backgrounds at several spatial scales [36, 37], yet crabs reared on various backgrounds all converge onto a uniform green-brown pattern as they age [38]. Tested alongside a range of juvenile patterns in a screen-based search task, this putatively generalist strategy proved most difficult to detect against natural backgrounds, suggesting that it provided better overall protection than the juvenile patterns, at least against human observers [38].

The extent of heterogeneity in natural environments, where animals experience continuous variation in both the number and proportion of different microhabitat types, as well as in their visual similarity [39], cannot be fully captured by experiments with a simple dichotomous set-up, in which prey occur on one of only two distinct patch types. A more sophisticated method, testing targets continuously varying in several properties, suggests that optimal camouflage can be achieved by adopting the most probable features of the scene, for both colour and pattern [40]. Yet so far this approach has been limited to the relatively simple, small-scale patterns of tree bark. Alternatively, machine learning tools, combined with genetic algorithms enabling evolution of target appearance, can home in on the most effective camouflage patterns from a much wider spectrum of possibilities [41, 42], but the resulting features are difficult to interpret and relate to background information.

Here, rather than testing generalist patterns between two contrasting patch types, we focus on a more realistic scenario, in which prey animals can either match one of several microhabitat patches from a natural scene, or resemble the average colour of the environment as a whole. Existing experiments have also been restricted with regards to background and target features. Most are based on variation in a single property of the background appearance, usually pattern size [21–23] (but see [44] for more naturalistic stimuli), despite evidence that colour and luminance are important for the optimisation of camouflage [40] and can affect the benefits of generalising [23]. Similarly, while the relative scale of the target and background patterning is known to affect target survival [24], all empirical investigations of generalist versus specialist camouflage have used relatively small targets in close proximity to the observer. Compounding the problem, most experiments testing specialist versus generalist camouflage strategies have been carried out on computer screens [22–24], or at best in artificial environments with controlled conditions [21, 44]. In most cases, visual search is therefore limited to a small area directly in front of the observer. Real-world search tasks in which volunteers look for camouflaged targets in the field have been used to test the effectiveness of other camouflage strategies [8, 45] and this method can similarly be applied to test background-matching strategies. Our study addresses these limitations and extends the relevance of work on compromise camouflage to more naturalistic scenarios, by testing its effectiveness in the field, with relatively large prey targets, and focusing on colour. Volunteers searched in two natural environments (farmland and woodland) for two-dimensional model hares painted to represent microhabitat specialist and generalist camouflage strategies. We then replicated this experiment in an online game, to disentangle the relative contributions of spatial scale and search scenario, in the field or on a screen, in explaining our results.

## Results

### Generalist and specialist strategies in the field

Volunteers were tasked with finding targets that adopted a generalist strategy (matching the average colour of the whole visual scenes), or that specialised on one of four microhabitat types (grass, leaf litter, bramble/ivy/other dark green vegetation, and bracken/other dried vegetation [hereafter referred to as bramble and bracken respectively, for simplicity]). Across 39 trials with 780 targets in total, 80 model hares (10.26 %) were not detected by volunteer participants, suggesting that the search task was relatively difficult. The maximum distance from which targets were visible varied between 30 and 200 m, with an average of 57 m across all positions, yet average detection distance by participants was 27 m.

**Fig. 1:**
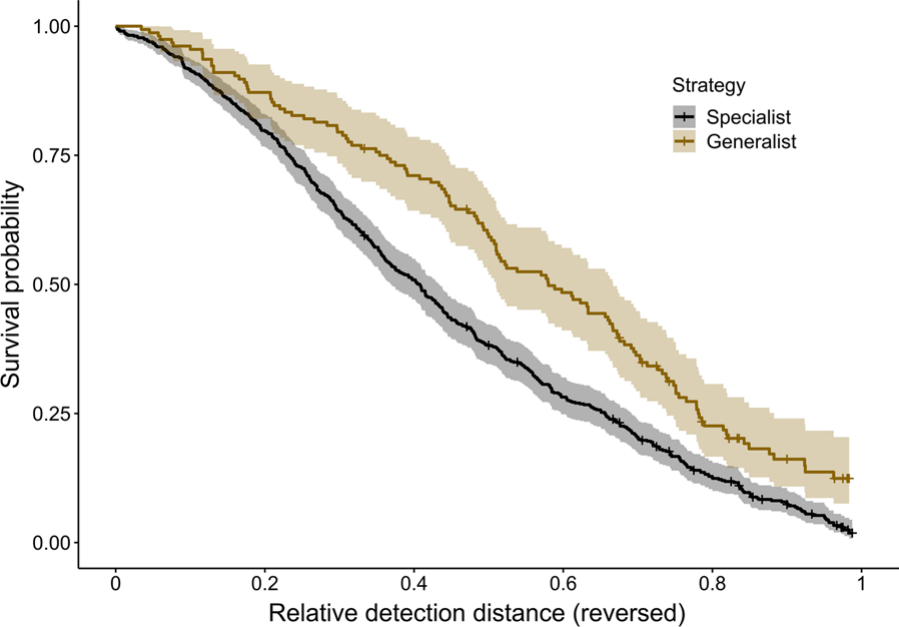
Survival probability of generalist and specialist models in the field. Distance is plotted as the inverse of relative detection distance: increasing values represent movement of the participants towards the model from the theoretical maximum viewing distance, so correspond to smaller detection distances. The shaded lines represent 95 % confidence intervals, and crosses indicate censored data (missed targets). The minimum distance at which participants could approach targets varied along the route, so the relative detection distances for missed targets reflect how close participants could ever get to those targets

There was no effect of presentation order (coxme, order: χ^2^_1_ = 0.342, p = 0.559) nor any significant interactions with habitat or difference between habitats affecting detection risk for targets of any type (coxme, habitat*weather: χ^2^_1_ = 0.004, p = 0.948; habitat*strategy: χ^2^_1_ = 1.994, p = 0.158; habitat: χ^2^_1_ = 1.862, p = 0.172). Across both habitats, there was no interaction between weather and camouflage strategy (coxme, strategy*weather: χ^2^_1_ = 0.558, p = 0.455), or any effect of weather conditions on detection risk (coxme, weather: χ^2^_1_ = 1.727, p = 0.189), but there was a clear effect of strategy (coxme, strategy: χ^2^_1_ = 37.779, p < 0.001): generalists were detected at shorter distances than microhabitat specialists (hazard ratio for generalists versus specialists, HR = 0.543, confidence interval (CI) = 0.443–0.667, z = − 5.84, p < 0.001; Fig. 1), suggesting better camouflage efficacy.

Survival analyses considering the specific colour of the hare models (two generalist paints, eight specialist paints) rather than overall strategy revealed a significant interaction between colour and habitat (coxme, colour*habitat, χ^2^ = 31.214, df = 9, p < 0.001), suggesting that the performance of different targets varied between the farm and woodland sites. Separate analyses for each habitat confirmed that detection risk varied between target colours (coxme, colour, in the farm: χ^2^ = 50.502, df = 9, p < 0.001; in the wood: χ^2^ = 81.268, df = 9, p < 0.001). However, planned comparisons between the colours showed that generalist types still performed best overall. In the woodland, one generalist colour (“Florentine Dream”) survived better than, or similarly to, all other colours, while the other (“Wagon Train”) was outperformed only by the other generalist and one grass specialist (“Pressed Olives”, HR = 0.414, CI = 0.248–0.690); in the farm, both generalist colours had higher or similar survival probability to all other colours (Additional file 1: Tables S1).

### Generalists and specialists in a computer-based scenario

**Fig. 2:**
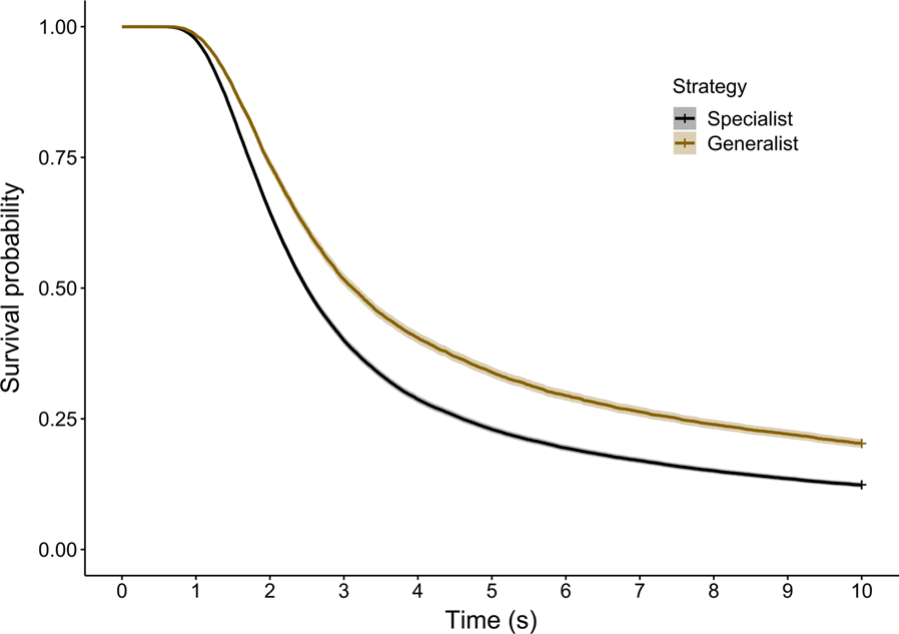
Survival probability of generalist and specialist models in the online experiment. Crosses indicate censored data (targets not found before time out), and the shaded lines represent 95 % confidence intervals

As in the field trials, generalist targets were more difficult to find than microhabitat specialists in the online experiment (coxme, strategy: χ^2^ = 1141.3, df = 1, p < 0.001; HR = 0.679, z = -32.13, CI = 0.663–0.695 for generalists versus specialists; Fig. 2). There was also a small but significant effect of slide number, with participants detecting targets faster as they became more experienced (coxme, slide number: χ^2^ = 845.79, df = 1, p < 0.001; HR = 1.023, z = 28.45, CI = 1.021–1.024). Repeating the analysis with hare colour instead of strategy confirmed that no specialist target type performed better than the generalist targets (coxme, colour: χ^2^ = 3619.5, df = 4, p < 0.001; HR = 1.525 [CI = 1.481–1.570], HR = 1.430 [CI = 1.388–1.473], HR = 1.062 [CI = 1.031–1.095], HR = 2.229 [CI = 2.164–2.296], for bracken, bramble, grass and leaf litter specialists respectively, relative to generalists; Additional file 2: Fig. S3).

### Effects of background similarity and structure on generalist survival

**Fig. 3:**
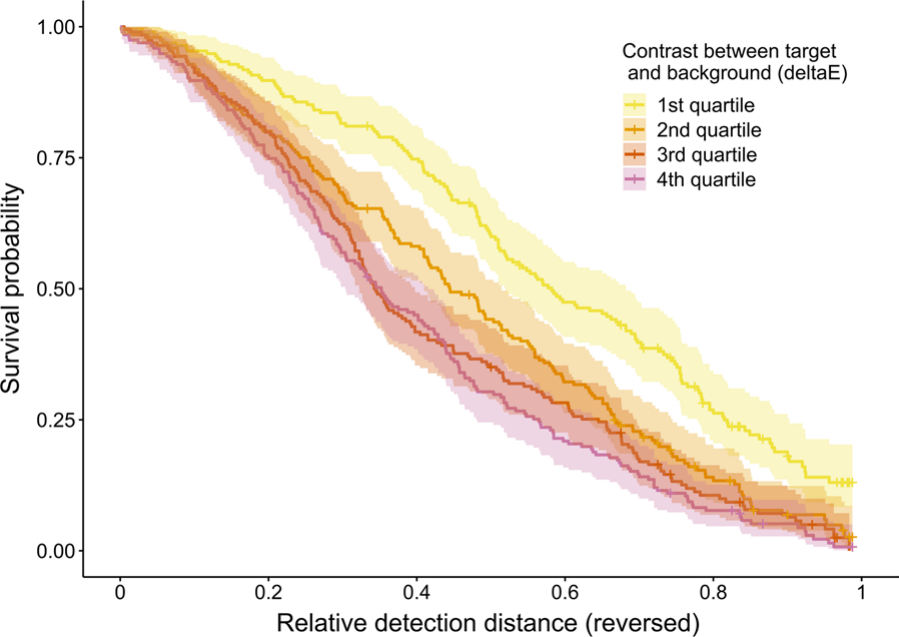
Survival probability of models based on contrast between targets and mean background colour. Background area is defined as the near zone surrounding the model hare, based on photographs taken 10 m away from the model. Contrast (∆E) values are split into quartiles for plotting purposes, with the smallest colour differences in the first quartile. Detection distance is plotted as the inverse of relative detection distance: increasing values represent movement of the participants towards the model from the theoretical maximum viewing distance, so correspond to smaller detection distances. Crosses indicate censored data (missed targets), and the shaded lines represent 95 % confidence intervals

To test the importance of the colour difference between targets and the specific backgrounds they were seen against in determining detection by humans, we re-analysed the field experiment data, replacing camouflage strategy with quantitative measures of the distance in CIELab space (∆E) between the targets and areas of the natural scenes, as explanatory variables in a series of survival models. Whether measured from images taken 10 or 30 m away from the model hares, considering the average colour of the whole image or a restricted near zone around the target, ∆E was always a significant predictor of detection risk, with greater differences between the models and backgrounds increasing detection risk (Table 1). Model comparisons using Akaike’s Information Criterion (AIC) indicated that all models including ∆E performed similarly or better than the model with strategy (specialist or generalist) as an explanatory variable. Measuring ∆E between targets and the near zone band of background around them provided the best predictors of target detection, particularly when the images were taken closer to the target (Table 1; Fig. 3), suggesting that contrast between the model and the area immediately around it, rather than the whole visual scene, was most important in determining detection risk.

**Table. 1:** Effects of colour differences between targets and backgrounds on detection risk in survival models

Explanatory variable										
Colour difference metric	Area	Distance from model	z	HR		CI		χ^2^_1_	p	AIC
∆E (CIEDE2000)	Near zone	10 m	8.56	1.064		1.049–1.080		74.691	< 0.001	7882.001
∆E (CIEDE2000)	Near zone	30 m	7.98	1.056		1.042–1.071		63.803	< 0.001	7891.923
∆E (CIEDE2000)	Whole image	10 m	7.44	1.054		1.039–1.068		55.802	< 0.001	7901.507
∆E (CIEDE2000)	Whole image	30 m	6.39	1.046		1.032–1.061		41.093	< 0.001	7916.372
Strategy	–	–	− 5.84	0.543		0.443–0.667		37.779	< 0.001	7919.648
Null	–	–	–	–		–		–	–	7958.985

Models are ranked by increasing AIC, including the model testing the effect of strategy (generalist/specialist) for comparison. Photographs taken from two distances away from the model (10 m/30 m) were used to measure coloration in different areas (near zone around the hare model/whole image), and differences between hares and backgrounds were measured as ∆E (CIEDE2000 formula). Hazards ratios (HR) > 1 indicate that increasing difference between models and background areas increases detection risk. The HR for the Strategy model corresponds to decreased risk for generalists compared to specialists. The null model includes the same random effects as all other models, but no strategy or colour difference variable

**Fig. 4:**
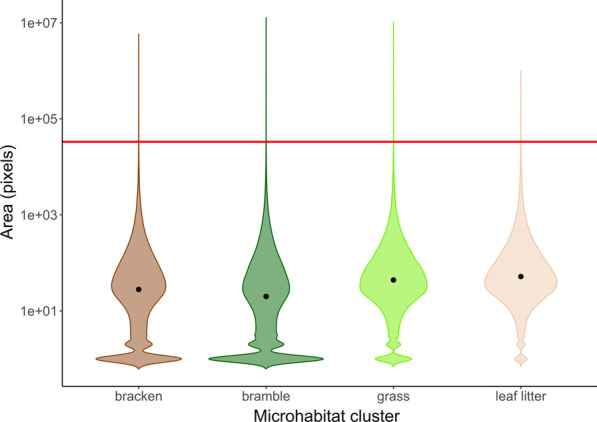
Area occupied by clusters representing each of the four selected microhabitat areas. Results represent NaïveBayes clustering of scaled images of all target locations, taken 10 m away from the target, with a 70 mm zoom. Median values for each microhabitat cluster are shown by the black points; the red line indicates the mean area occupied by the hare-shaped target in the same images

Together, the importance of matching the average colour of the immediate surroundings of the targets to reduce detection and the strong performance of the generalist types suggest that, in our experiments, the targets were rarely seen against a single microhabitat type. We verified this observation by clustering images of the natural scenes around the targets into patches corresponding to each of the four microhabitat areas. The target was substantially larger than most microhabitat patches in these scenes, making it very unlikely that it could be surrounded by a single microhabitat type: patch size was highly skewed, but median values were orders of magnitude smaller than the visible area occupied by the hare models (median area_bracken_ = 28, area_bramble_ 20, area_grass_ = 44 and area_leaf litter_ = 52 pixels; mean hare area = 33,229 pixels, st. dev. = 3712; Fig. 4). Re-analysis of the field data shows that microhabitat specialist targets did survive better when seen against backgrounds that contained a larger proportion of the matching microhabitat type,  confirming the suggestion that their poor performance is linked to the structure of the background scenes, and the relative scale of the targets and microhabitat patches. While the proportion of the area in the whole visual scene occupied by the same microhabitat as represented by the target had no effect on detection risk (coxme, χ^2^_1_ = 0.254, HR = 0.888, CI = 0.558–1.412, z = − 0.5, p = 0.615), there was a significant effect when this proportion was calculated for a restricted near zone around the target, such that targets surrounded by a greater proportion of the microhabitat they represented were less likely to be detected (coxme, χ^2^_1_ = 7.376, HR = 0.546, CI = 0.350–0.851, z = − 2.67, p = 0.00661).

## Discussion

For our relatively large targets, differing in colour, we found that a ‘generalist’ global background-matching strategy, resembling the average colour of the visual environment, was more successful in evading detection by human volunteers in natural situations, than specialists matching individual microhabitat elements. While the number of participants in the field trials is small, the generalist advantage is robust to re-analysis with subsets of the data, limiting results to participants who visited both field sites and excluding a colour-blind participant, and is reproduced in a different search scenario by the computer-based experiment, with a much larger sample size.

By contrast, previous computer experiments with targets varying in colour and luminance generally conclude that specialising on one background patch incurs a lower detection risk, or at least performs no worse, than adopting a compromise strategy [23]. Concordance between our results in both field and screen scenarios suggests that this divergence is not due to the specific nature of our field-based search task, in a three-dimensional environment. However, a number of important factors differentiate our present experiments from earlier studies, and taking these into consideration suggests that our results do fit in with expectations based on existing theoretical and empirical work. Most obviously, the natural scenarios we used differ from typical experimental set-ups, in which targets are shown either on a heterogeneous background consisting of sharply-defined distinct colour patches [23] or sequentially against different backgrounds [22]. A generalist advantage in our study is less surprising than it might seem at first, since there is little variation in colours in the temperate natural environments considered in our experiments. The visual scenes are mainly limited to shades of brown and green, so targets matching the average colour of entire scenes are still relatively close to the colours representing microhabitat specialists [see Additional file 3]. As similarity between backgrounds is a key predictor of success for compromise camouflage strategies [14, 21], the type of natural backgrounds used in our experiments stands to benefit a more generalist strategy; in artificial or natural environments with more contrasting microhabitat patches, we would predict that the global match generalist target type would be less successful. Nonetheless, while many of the colours across treatments here are quite similar, the clear contrast in survival in both experiments show that the differences still have a strong influence on detection.

The natural backgrounds used here display more realistic variation in the spatial scale and distribution of microhabitat patches, compared to more artificial experimental systems, which may also reduce the effectiveness of microhabitat specialist strategies. Heterogeneous backgrounds, in combination with the use of visual search images by predators, are thought to favour variation in prey coloration and polymorphisms [24, 46], based either on visually complex backgrounds making multiple phenotypes equally cryptic, or on patchy backgrounds facilitating specialisation on different microhabitats [47]. In a classic experiment, predation by blue jays selects for different strategies in a population of artificial moths, depending on the relative scale of the targets and background patches [24]. In disjunct and mottled treatments, where the background patches were fifteen times larger or equal to the target moth size respectively, specialists resembling either light or dark patches were favoured. By contrast, more generalist patterns evolved on the speckled backgrounds, with patches roughly twelve times smaller than the moths. In our natural environments, the distribution of colour patch types is much less controlled than in typical experimental set-ups. There is also substantial overlap between patches (for example, areas of intermingled leaf litter and grass), and our targets are much larger than prey items used in any previous work on this topic. Avoiding detection in our experiments is critically linked to low colour differences between the targets and their immediate backgrounds, so generalists must be better camouflaged against their immediate surroundings than the microhabitat specialist types. Our analyses suggest that, in our chosen natural environments, microhabitat patches are typically smaller than our target, so an animal the size of our model hare would not be reliably found against a single microhabitat patch type, and would in fact be more likely to be viewed against a patchwork of microhabitats, with an average colour close to that of the whole visual scene. This situation closely mirrors, on a larger scale, that of the artificial moths seen against the fine-grained backgrounds in Bond and Kamil’s experiments [24]. Our findings are also compatible with those of [40], who found that optimal camouflage could be achieved by adopting the most probable features of the background (in terms of colour, pattern and luminance), at the scale of the target. In our natural scenes, it is likely that samples of the backgrounds, taken at the size of the target hare model, would be biased towards an average colour close to that of the whole visual scene, rather than that of any given microhabitat patch. This scenario likely parallels many natural instances of camouflage in real, larger animals (e.g. many ungulate mammals), where body size will often exceed that of the immediate colour patches around them. This may be one reason why many of these animals tend to adopt more uniform or similar hues of brown or grey, even if more specific associations between coat colour and habitat coloration for background-matching are not supported in even-toed ungulates [48].

The critical role of prey position relative to microhabitat patches in determining optimal camouflage strategies is demonstrated by the behaviour of animals with colour-changing abilities. While octopuses [49] and twig-mimicking peppered moth *Biston betularia* caterpillars [50] appear to specialise on certain elements of their habitat when confronted with heterogeneous backgrounds, other animals opt for compromise phenotypes, depending on microhabitat patterns. Experiments with tree frogs *Hyla japonica* found they adopted an intermediate coloration when resting on checkerboard patterns [51], but check size was much smaller than the frogs themselves, so each frog always found itself on a patchy background. Earlier work with spring peepers *Hyla crucifer* suggests that frogs can select different strategies based on pattern size and the position of their body relative to pattern patches: on striped black and white backgrounds, with stripes wider than the frogs, most animals chose to rest inside a single stripe, and adopt matching coloration, but those resting across stripes displayed a compromise pattern of intermediate lightness, as did active animals moving across stripes [52].

While the use of natural environments and larger targets increased realism in our experiments, our uniform targets were relatively simple in design. We focused on the effect of colour-matching, as previous tests of compromise strategies suggest that humans may be more sensitive to colour rather than pattern differences [23]. With uniform targets, we cannot evaluate the relative importance of pattern versus chromatic and achromatic contrast to the background in these experiments, but our results do demonstrate a strong effect of colour difference on detection probability: a greater colour difference between the targets and the backgrounds they were seen against was significantly associated with increased detection risk, despite a substantial mismatch in pattern texture between complex natural scenes and uniform targets. The colour difference between targets and their immediate surroundings was also a better predictor than the difference from the entire visual scene, implying that local contrasts were most important in facilitating or hindering detection. The greater sensitivity of humans to colour rather than pattern mismatches has led to the suggestion that it may be more difficult for generalist strategies to succeed in environments varying in colour rather than in pattern [15]. However, in this case, since the targets were relatively large compared to background patterning, the importance of local colour matching actually favoured generalist targets, which better matched the average colour and luminance of the patchwork of microhabitats surrounding the targets.

Using human observers, rather than more natural predators, limits the interpretation of our findings with regards to specific prey colours, yet these experiments do highlight some general principles for camouflage in nature. The importance of the relative scale of prey and background elements suggests that, in all but the most uniform of habitats, larger animals, at least sufficiently large to exceed the typical size of a microhabitat patch, may benefit from generalist coloration over microhabitat specialisation. Evidence from predation experiments with birds, computer-based search tasks, and artificial evolution using neural networks also suggests that complex backgrounds make detection tasks harder, and generally impose less stringent requirements for accurate background-matching [53–55], making compromise coloration yet more likely to provide some protection in natural situations. In particular, screen-based experiments explicitly testing combinations of background heterogeneity and complexity (in terms of background element shapes) found that higher background complexity enhanced the survival of generalist targets, when the visual difference between backgrounds was low [56], as is the case in our experiments. Including variation in pattern in future studies of detection risk for large targets in the wild would further increase the relevance of these insights. Matching pattern statistics between the targets and backgrounds is important for optimising camouflage [40], and other pattern-based strategies may independently enhance crypsis. Disruptive camouflage, in which the outline of the prey is broken up by high-contrast markings [6, 57], may provide some form of generalist camouflage, less dependent on the specific visual environment than background-matching strategies [15], as seen in putatively-generalist striped morphs of pygmy grasshoppers (*Tetrix subulata*) [43], shrimp (*Hippolyte obliquimanus*) [58] and jumping spiders (*Anasaitis* sp.) [59]. Patterns such as disruptive markings could also work in concert with compromise or specialist background-matching strategies, and the interplay between different colour and pattern-based strategies deserves investigation. Finally, the relative size of prey and background pattern elements is likely to influence the benefits of different pattern types, such as the effectiveness of edge disruption for disruptive coloration [21]. Screen-based experiments with photographs of grasshopper morphs suggest an interaction between colour pattern and body size in determining detection risk [60], within a fairly small naturalistic size range of targets, and effects could be greater in larger targets. It would be useful to test how patterning, whether matching the background scale or specifically designed to be disruptive, affects detection of larger targets similar to those used in this present study. This would help complete the picture to better understand how size, coloration strategy, pattern type, background complexity, heterogeneity, and relative pattern scales interact to determine optimal camouflage strategies.

Going forward, our experiments also provide further validation of widely-used computer-based tests of camouflage theory. In this and previous studies, detection tasks on computer screens have proved useful tools, with key advantages being easier acquisition of large sample sizes [23] and circumventing difficulties associated with producing physical targets. Combining our field results with the online experiment confirmed that the global-matching generalist solution was genuinely advantageous, even when not limited to selecting available paint colours: in the screen-based task, there was no difference in the colour matching success of different target types, as these are directly based on the mean RGB values for each natural area. Several studies have previously found comparable results between field trials with wild predators and human detection experiments on screens [61, 62], suggesting that computer-based experiments can reveal trends relevant to camouflage in real-world situations, while any discrepancies in results can expose potentially interesting differences between wild predator species and humans, such as variation in attention to luminance versus pattern cues in humans and birds [40, 55]. However, few studies have tested the same predator in different scenarios, making it difficult to disentangle effects of differences in perception and processing from effects of changes in the nature of the task, particularly between two-dimensional screens and three-dimensional natural environments, which offer much greater freedom of movement for searching predators. One exception is work exploring the detectability of pygmy grasshoppers (*Tetrix subulata*), which found that different morphs were detected in similar proportions by humans, whether searching for them in the wild or viewing photographs on a screen, and that their performance was related to morph frequency and survival in the wild [43]. Supporting their findings with much larger targets, the agreement between the results of our field-based and online search tasks increases confidence in the relevance of computer-based experiments for testing questions relating to camouflage, at least from a human perspective.

## Methods

### Model design and study sites

Field trials were carried out between 26th February and 17th March 2020, on two sites in Cornwall (UK), a woodland (Cosawes wood, 50°11’51” N, 5°7’33” W) and farmland managed for conservation purposes (Trelusback farm, 50°12’5” N, 5°12’26” W).

**Fig. 5:**
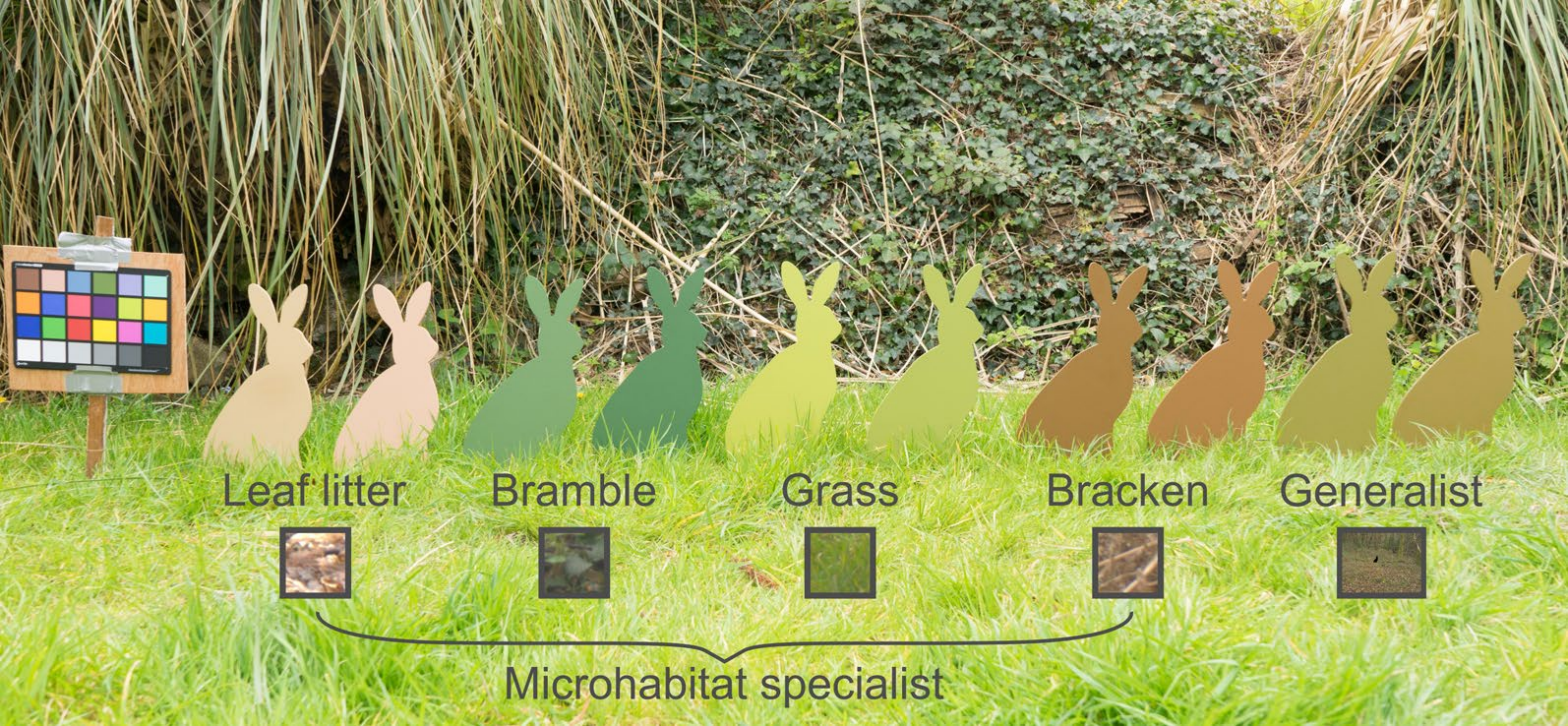
Model hares of all 10 paint colours chosen. In pairs from left to right, they represent specialist (leaf litter, bramble, grass, bracken) and generalist strategies. The small squares beneath the labels provide examples of the selections the models were designed to match, including a whole image scene for generalists

Targets were designed to be the shape and size of a hare (40 cm tall × 26 cm wide), chosen as an easily-recognisable natural shape, much larger than typical targets in detection experiments, but small enough to ensure the task remained difficult. These targets were laser-cut from 6 mm thick birch plywood (The Grain Ltd. Liskeard, UK), and inserted into the ground with a wooden spike. Each model was painted a uniform colour, representing microhabitat specialist and generalist camouflage strategies, based on real colours found in the natural environments in which the targets would be seen (Fig. 5).

### Photography and image analysis

Calibrated photographs of field locations were used to identify specialist and generalist colours, and later to analyse colour differences between the models and the exact natural backgrounds they were seen against. All photographs were taken with a SONY A7 camera fitted with a 28–70 mm lens (SONY, Tokyo, Japan) with fixed settings (RAW, f.8, ISO 400, white balance set to cloudy), in diffuse lighting conditions. Each image included a Classic ColorChecker® chart (X-Rite Inc., Grand Rapids, USA) to enable normalisation with respect to light levels and provide a scale bar. Camera calibration and all image analyses were carried out with the Image Calibration and Analysis toolbox-MicaToolbox [63, 64] in ImageJ [65]. Cone catch models for human vision were created based on photographs of the colour chart, following custom plugins in the toolbox. Images were linearised, normalised and converted to coordinates in human CIE XYZ space, and from there into the CIELab colour space [66], a representation of human colour discrimination, approximating a perceptually uniform colour space, and widely used to assess human colour perception [67, 68]. CIELab coordinates account for achromatic and chromatic information, defining a colour along three axes, representing lightness (L) and colour, from green to red (a) and blue to yellow (b).

The hare-shaped targets were designed to represent camouflage strategies matching a specific microhabitat type in either habitat (“microhabitat specialist”), or adopting a compromise, global-matching solution, resembling the average colour of the visual landscapes (hence known as “generalist”). To identify appropriate colours in our field sites, we photographed likely locations for targets in each area (N_wood_ = 22, N_farm_ = 20) in January and February 2020. In each habitat, two types of common natural elements in the environment were selected as the basis for targets of microhabitat specialist camouflage: leaf litter and bramble/other dark green shrubs in the woodland, grass and bracken/other dried and brown vegetation in the farmland. In each image, transformed to CIELab space, square selections representing 10 cm^2^ areas of the two relevant specialist elements were taken using the rectangle selection tool. Generalist colours were based on the average colour of the entire visual scenes in these images, across both farm and woodland, excluding colour standards, sky and large man-made objects.

Ideal target colours were then compared to 578 paint samples from the Valspar® range (Valspar, Wokingham, UK). Sample cards were photographed and analysed using the same technique described above. We first identified paints whose CIELab values fell exclusively within the range of values for a single set of target colours (grass, bracken, leaf litter, bramble or whole images). If more than two colours fit this criterion, we selected the best two matches in terms of distance in CIELab space (∆E) between the paint colours and the median target colour, provided that the paints did not also match the colours of any other target groups just as closely. ∆E was calculated according to the CIEDE2000 formula [68–70], an adjustment to Euclidean distance officially adopted by the Commission for International on Illumination (CIE) in 2001 [71], which accounts for some remaining perceptual non-uniformity in the CIELab space and, under appropriate viewing conditions [72], better predicts colour discrimination by humans than previous formulations [69, 73, 74]. This protocol yielded a total of ten paint colour selections, two each per type of microhabitat specialist or generalist treatment (Fig. 5; Additional file 4:Tables S6). Samples from the chosen paints were then painted onto plain birch plywood to check that the actual paints themselves fulfilled these criteria. Painted squares were photographed outdoors and analysed as above, and ∆E values (CIEDE2000) between the paint values and target selections were calculated (Additional file 4:Tables S6). We subsequently analysed the colour difference between the painted hare models and all natural areas of interest again after the field trials were carried out, based on photographs of the models *in situ*, to verify that the microhabitat specialist targets were indeed best matched to the areas they were supposed to represent [see Additional file 3].

### Field trials

A total of 24 volunteers, aged 19 to 47 (N_female_ = 17, N_male_ = 7) participated in the experiments; 15 of them performed the search task in both the wood and farm, while the others were only able to visit a single location, yielding a total of 39 trials (N_farm_ = 18, N_wood_ = 21); no further data collection was possible due to restrictions linked to the Covid-19 pandemic. Of those who completed both trials, 12 out of 15 participants were tested in the farm before the wood. A simple colour vision test using Ishihara plates (24-plate edition, Kanehara Trading Inc., Tokyo, Japan [75]) was carried out in the field prior to the search tasks – a single participant did not pass the screening, and subsequent analyses were carried out with and without their trials [see Additional file 5]. Volunteers were recruited by word of mouth, compensated for their time according to the university guidelines for participation payments to research volunteers, and provided written consent for their results to be used in this project.

At each field site, both woodland and farmland, 20 model hares (two of every colour: eight specialist colours and two generalist colours) were set out at fixed positions, a minimum of 30 m apart, either side of a predetermined path. Models were placed in a random order at the start of each day of field trials, then moved along by one position after each participant had completed the trial, so that every volunteer experienced a different combination of model colours and background locations. Along each transect, an equal number of targets were placed facing left or right, in a randomised order. Each one was visible from a minimum of 30 m away, as the volunteers walked along the route, but, due to differences in topography, path layout and the presence of occluding vegetation, the maximum and minimum detection distances for targets varied between positions; these distances were recorded and used to standardise detection distance in subsequent analyses. Volunteers were tested individually, with an experimenter walking behind, to guide them without influencing their search. They were encouraged to walk at a comfortable pace and search as they moved, without stopping to scan the scene; when they spotted a model, they stopped and the experimenter recorded the distance between the subject and the model (detection distance), using a laser range finder (MLR01, Tacklife, USA). Participants also took a photograph of the target from where they stood, using the same equipment and settings as the photography for image analysis, to preserve a record of the viewing conditions. Based on these images, weather conditions were later classified as overcast or sunny for each detection event; experimenters made a note of conditions when targets were missed.

### Analyses of target camouflage

To analyse landscape coloration in the specific locations in which targets were seen by volunteers, a new set of field photographs was taken at the end of the experiment. Each target location was photographed with a model hare in place (painted pink - “Pinkberry Passion”, R65E - to stand out against the natural backgrounds), from 10 and 30 m away, with the same camera equipment and settings as above, and a 70 mm zoom lens. Images were scaled to 0.8 and 0.3 pixels/mm respectively and processed and transformed to CIELab space as described above. In each image, we selected the hare target using the colour threshold tool in ImageJ, and, following methods in [76], defined two further areas for analysis: the immediate surrounds, a band the width of the hare target height (40 cm) around the target, and the whole visual scene, excluding the skyline and large man-made structures (see  Additional file 6: Fig. S5), from which we measured mean CIE Lab values. A narrow band of pixels (4 and 2 pixels wide for 10 and 30 m images respectively) was excluded around the outline of the model to ensure that no model pixels were mistakenly included in the background zones. The painted targets of different colours used in the experiment (N_model_=10) were also photographed and analysed in the same way: a large area in the centre of each hare was selected using the polygon tool in ImageJ, from which the average colour was extracted. Colour differences between every target type and all background areas were once again measured in ∆E (CIEDE2000).

Photographs of the pink model hare *in situ*, taken from 10 m away, were also used to quantify the relative size of the hare-shaped targets and the areas resembling each microhabitat (grass, bracken, bramble and leaf litter) in the visual scenes. Using the MicaToolbox Quantitative Colour Pattern Analysis (QCPA) framework [64], images in human CIE XYZ space (N = 40) were first smoothed with the receptor noise limited (RNL) ranked filter tool (weber fraction = 0.05, weber fraction for luminance = 0.1, kernel radius = 3, falloff = 2 and 3 iterations), to facilitate clustering. They were then transformed to CIELab space, and the built-in Naïve Bayes classifier tool from the MicaToolbox QCPA [64] was applied to segment the image (excluding the target and exclusion zone), based on the mean and standard deviations of the four microhabitat areas initially selected to design the targets. This process assigns each pixel to a microhabitat cluster, based on similarity to the colour of that microhabitat type. Finally, cluster particle analysis was used to measure the area of every individual patch belonging to each cluster, and the proportion of the total area occupied by pixels corresponding to each microhabitat was calculated, for both the whole image and near area band around the targets.

### Online experiment design

An online game was created to replicate the field trials in a computer-based search task, using custom Javascript code, adapted from code previously-used in similar citizen science experiments [77], and made freely available to play on internet browsers (at: http://fieldhares.sensoryecology.com; see Additional file 2: Fig. S1). Images were prepared from photographs of the pink model hare, facing both left and right, at each field location, taken from 30 m away with a 70 mm lens, and transformed to human XYZ space as described above. These images were converted to 24-bit RGB colour images in ImageJ, using the ‘Make Presentation Image’ plugin in the Image Calibration and Analysis toolbox-MicaToolbox [63, 64] to move from CIE XYZ to sRGB space, with a maximum brightness value of 1 and a power of 0.42, corresponding to the non-linear transform for sRGB images. To choose RGB values for microhabitat specialist and generalist targets, the same regions of interest used to analyse the colour of natural areas in CIELab space (as described above) were applied to the new RGB images from each field location, and the average R, G & B values of each area across images was calculated (specialists: bracken RGB = 143,125,92, bramble RGB = 112,121,85, leaf litter RGB = 165,139,113, grass RGB = 132,148,78 & generalists: whole image RGB = 123,122,81). The next step was to ensure variation in the position of the target on the screen, and in the size of the target, mimicking the experience of volunteers in the field trials, who might not always see the target from the same distance. Every RGB image was manually cropped to generate three images 1920 × 1080 pixels in size (hereafter, small crop), and three images twice the size (3840 × 2160 pixels, large crop), where the hare model would appear smaller; in each of these, the model hare and the surrounding exclusion zone were selected and recoloured to each of the five treatment colours, generating a database of 2400 images in total (see Additional file 2: Fig. S2, for a representation of the image preparation process).

When playing the game, participants were shown a set of 20 slides, each with a single hare target to locate: each set included an equal number of backgrounds from the wood and farm environments, with no repeats, along with an equal number of target hares facing left or right, and of every colour, in a randomised order. To create variation in difficulty and maintain interest, 8 out of 20 slides featured small crop size images, where the hare was larger and thus easier to find, and the remainder were large crop images. Participants were given 10 s to find the target in each slide, and received feedback on their success. If they correctly clicked on the target, a ‘positive’ sound was played and a green circle appeared around the hare before the background faded away. By contrast, clicks in incorrect locations triggered a ‘negative’ sound, and, if the target was not located within the time limit, a red circle highlighted its position. The timing and position of all clicks, including misses in incorrect locations, were recorded.

### Statistical analyses

All colour matching analyses and statistical analyses were carried out in R, version 3.5.2 (“Eggshell Igloo”) [78]. The DeltaE function in the ‘spacesXYZ’ package [79] was used to calculate ∆E (CIEDE2000).

The effect of camouflage strategy on the probability of detection was tested using Cox mixed effects survival models, implemented with the package ‘coxme’ [80]. In all models for the field trials, detection distance was used as a measure of detection risk for the camouflaged targets. To account for variation in how far models at different transect positions could physically be seen, this distance was scaled as a proportion of the maximum possible viewing distance at each location, providing a measure of relative detection distance. To match typical survival model outputs, in which increasing time to capture indicates better survival, we then took the inverse of this relative distance, so that increasing values represent an increase in the relative distance participants walked towards the target model from its theoretical maximum viewing distance, before detecting it. The full survival model included strategy, habitat (farm or woodland) and weather, with their pairwise interactions, as well as presentation order, as fixed effects, with subject ID and position as random effects, to account for variation in ability between participants and in difficulty between specific sites along the transect. Model simplification using likelihood ratio tests was performed to determine the significance of fixed effects on survival probability. Final survival models were relevelled to provide a hazards ratio (HR) for the effect of being a generalist rather than a specialist, where an HR greater than 1 indicates that the probability of detection increases and an HR below 1 that it decreases with this strategy [81]. This analysis was then repeated, replacing the strategy variable with the specific paint colour applied to the models (two generalist colours and eight specialist colours), to check that testing for an overall effect of strategy did not mask the success of particular specialist colours. Where a significant interaction with habitat was found, separate models were then run for the farm and woodland in turn.

To further investigate the factors affecting detection risk, we performed two additional series of analyses. To test the effect of similarity between the colours of the targets and the areas they were seen against, the mixed effects Cox survival models described above were re-run with quantitative measures of colour differences, in ∆E, between models and different background areas (near zone and whole image) as explanatory variables. Their performance in explaining variation in detection risk was compared by computing the Akaike Information Criterion (AIC) [82] for each model; models with Δ_AIC_ values greater than 6 were considered to differ substantially in their explanatory power [83]. Finally, we tested whether the proportion of the scene visually similar to each microhabitat affected detection risk for the microhabitat specialist targets in the field. Survival analyses were re-run on the field data restricted to only microhabitat specialist target types, with the proportion of area occupied by the same microhabitat cluster as the target, in either the whole image or near area band, as a continuous explanatory variable. In all survival models, subject number and position were included as random effects, and the proportional hazards assumption was verified.

For the online game, data downloaded from the server on 15th February 2021 recorded a total of 2906 plays. Participants playing on mobile devices were excluded due to the small screen size, as were an additional three plays where results for only 19 out of 20 slides were recorded, leaving 2804 plays for analysis, with 1955 unique players. Similarly to the field trials, results were analysed with Cox mixed effects survival models, using the package ‘coxme’ [80]. The main model included strategy and slide number as fixed effects, with participant number and image ID, corresponding to its position in the field, as random effects, to account for variation in individual performance, including differences in the devices used to play the game, and variation in task difficulty based on the specific position of the target in the photographs. Target location relative to the centre of the screen has been shown to be a significant predictor of detection times in similar experiments [84], with more central targets easier to find [85], and the size of the target, determined by image crop size, was also expected to be important. Including distance from the screen centre and crop size in the model led to non-proportional hazards, so the model was instead stratified by crop size (large or small) and distance from the centre, discretised into quartiles, to account for these effects. A second model was then fitted with strategy replaced by hare colour. For all coxme models, for both field and computer results, we verified that the proportional hazards assumption was satisfied, using diagnostic plots and the cox.zph function in the package ‘survival’ [86], for the equivalent coxph models with no random effects, and, where possible, for coxph models with random effects included one at a time as frailty terms; deviations according to the cox.zph test were tolerated, depending on inspection of plots of the Schoenfeld residuals.

## Supplementary Information

**Additional file 1.** Survival probability for targets of different colours, in the field trials.

**Additional file 2.** Additional information for online experiment.

**Additional file 3. **Match between natural areas and target paint colours in field trials.

**Additional file 4.** Paint colours used in field trials.

**Additional file 5.** Field trial analyses, repeated with restricted datasets.

**Additional file 6.** Methods for analyses of target camouflage.

## Data Availability

The data and code supporting this publication are openly available on the Open Science Framework repository at: https://osf.io/6p2fw/ [87].

## Abbreviations

∆E: Distance in CIELab space
AIC: Akaike’s Information Criterion
CIE: International Commission on Illumination
CIEDE2000: Formula for color difference in CIELab space
CIELab: Color space defined by the CIE to represent human vision
HR: Hazards ratio
RNL: Receptor noise-limited
