# Supplementary material for: Generalist camouflage can be more successful than microhabitat specialisation in natural environments
Source: BMC Ecol Evol. 2021 Aug 3;21:151. doi: 10.1186/s12862-021-01883-w (PMC8330473; doi:10.1186/s12862-021-01883-w)
Supplement: Supplementary file 1 — Additional file 1. Survival probability for targets of different colours, in the field trials. [file 12862_2021_1883_MOESM1_ESM.pdf]

<u>Additional file 1:</u> Survival probability for targets of different colours, in the field trials

Supplementary Table 1: Hazard ratios (HRs) comparing the survival probability of specialist to generalist targets, by colour, and for each habitat. Confidence intervals (CI) excluding HR=1 indicate significant differences between colours.

a. Hazard ratios relative to Generalist 1 (“Florentine Dream”)

Colour	Habitat: Farm		Habitat: Woodland	
	**HR**	**CI**	**HR**	**CI**
AC	1.545	0.912 – 2.616	3.174	1.867 – 5.397
VP	2.300	1.369 – 3.863	4.769	2.856 – 7.963
CH	2.576	1.529 – 4.341	2.108	1.255 – 3.542
PO	1.514	0.899 – 2.552	0.961	0.552 – 1.674
CL	2.009	1.194 – 3.379	3.072	1.821 – 5.184
TC	2.720	1.612 – 4.591	4.714	2.822 – 7.874
HG	1.761	1.051 – 2.949	2.292	1.368 – 3.841
LG	4.258	2.543 – 7.128	2.534	1.507 – 4.260
WT	1.002	0.594 – 1.692	2.321	1.393 – 3.869

b. Hazard ratios relative to Generalist 2 (“Wagon Train”)

Colour	Habitat: Farm		Habitat: Woodland	
	**HR**	**CI**	**HR**	**CI**
AC	1.541	0.915 – 2.596	1.367	0.855 – 2.187
VP	2.294	1.375 – 3.827	2.054	1.296 – 3.258
CH	2.570	1.529 – 4.319	0.908	0.567 – 1.454
PO	1.511	0.907 – 2.516	0.414	0.248 – 0.690
CL	2.004	1.198 – 3.353	1.324	0.823 – 2.128
TC	2.714	1.628 – 4.524	2.031	1.279 – 3.226
HG	1.757	1.052 – 2.934	0.987	0.613 – 1.591
LG	4.247	2.543 – 7.088	1.092	0.678 – 1.756
FD	0.998	0.591 – 1.684	0.431	0.258 – 0.718
