# Supplementary material for: Generalist camouflage can be more successful than microhabitat specialisation in natural environments
Source: BMC Ecol Evol. 2021 Aug 3;21:151. doi: 10.1186/s12862-021-01883-w (PMC8330473; doi:10.1186/s12862-021-01883-w)
Supplement: Supplementary file 2 — Additional file 2. Additional information for online experiment. [file 12862_2021_1883_MOESM2_ESM.pdf]

Additional File 2: Additional information for online experiment

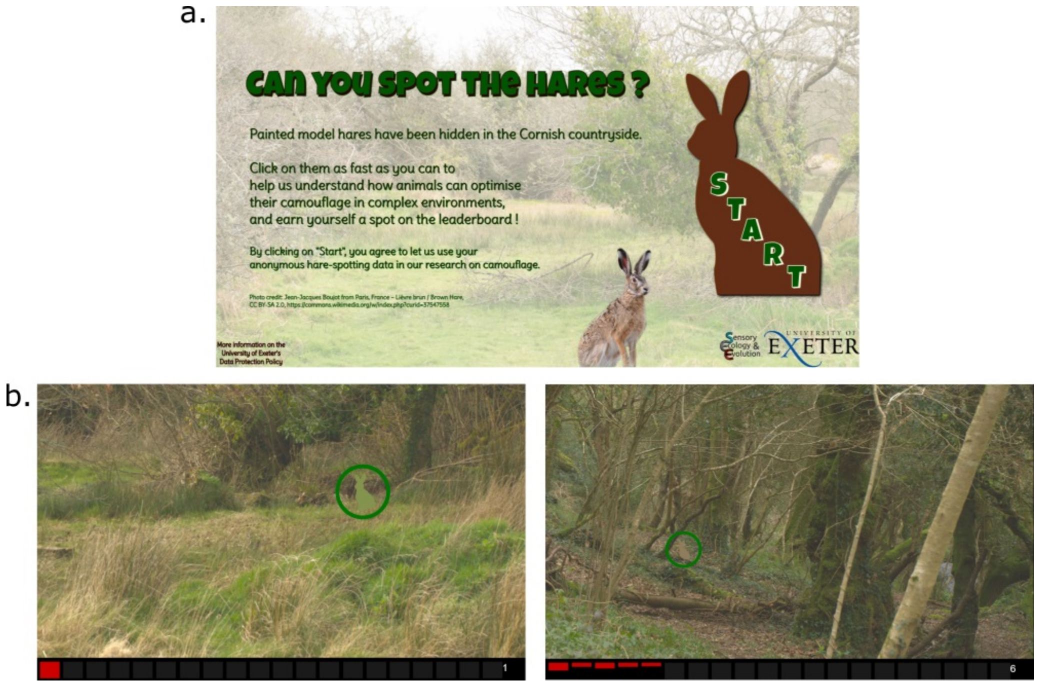

Supplementary Figure 1: Online game loading screen (a) and example slides (b), showing hare targets on images of different sizes and from the two habitats (farm, smaller crop size, on the left, and wood, larger crop, on the right). Green circles highlight the targets.

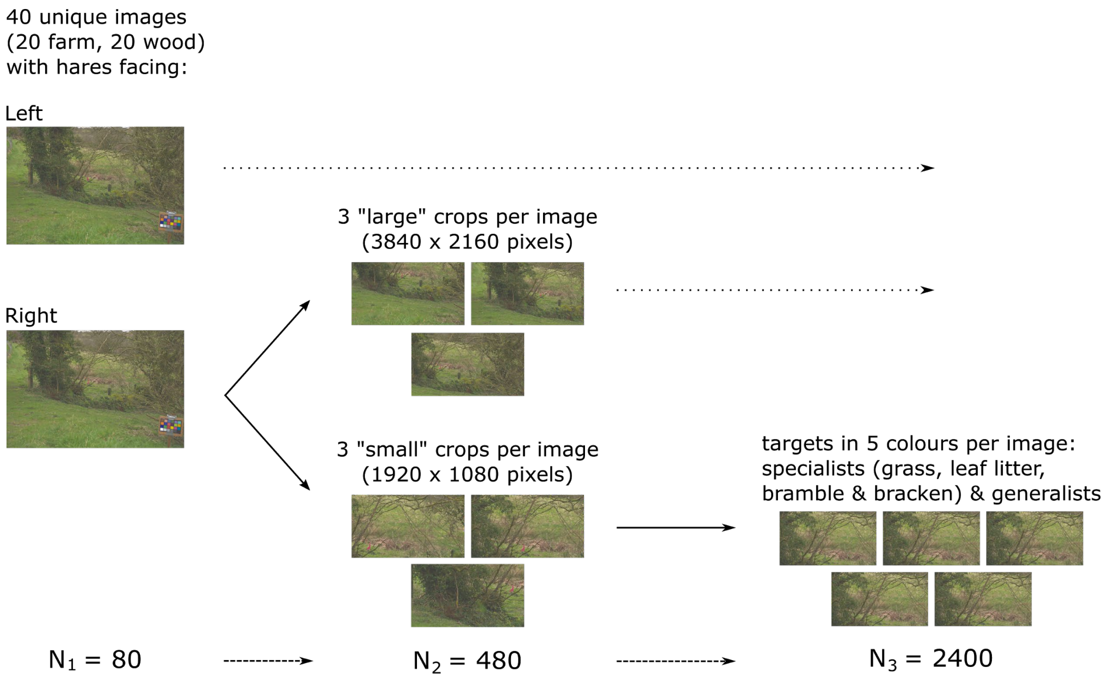

Supplementary Figure 2: Diagram representing methods for generating images for the online game.

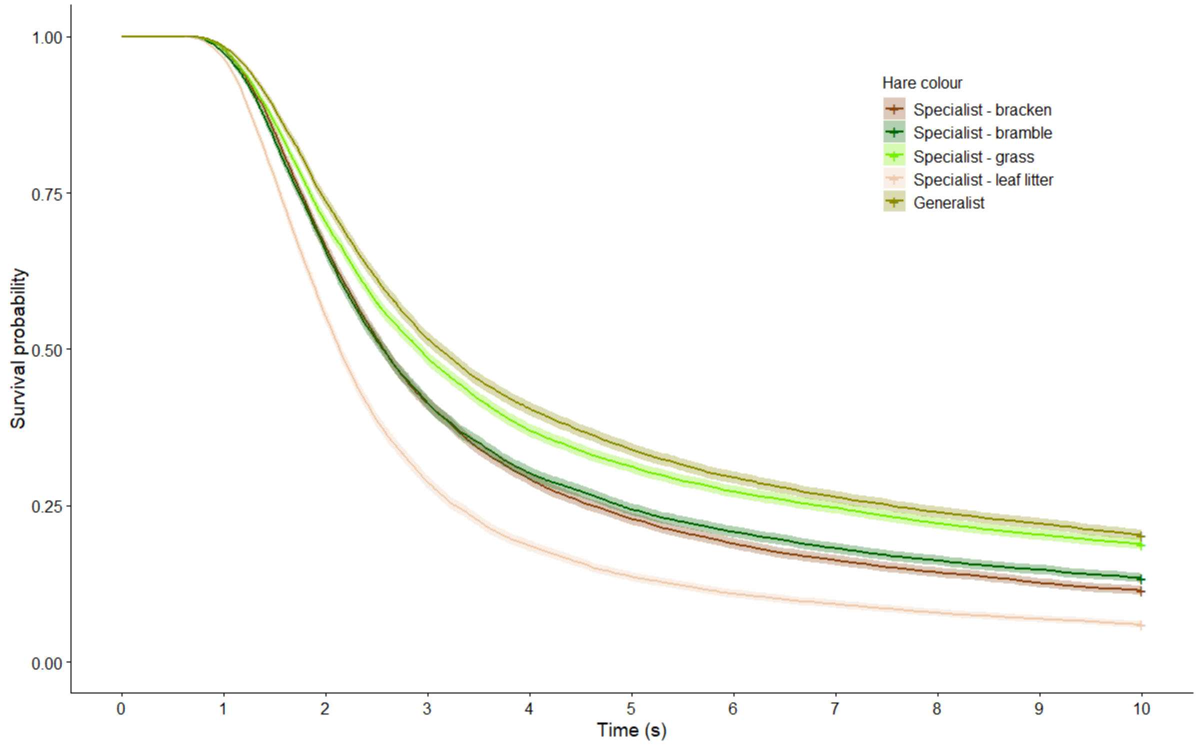

Supplementary Figure 3: Survival probability of targets of different colours in the online experiment. Crosses indicate censored data (targets not found before time out), and the shaded lines represent 95% confidence intervals.
