# Supplementary material for: Generalist camouflage can be more successful than microhabitat specialisation in natural environments
Source: BMC Ecol Evol. 2021 Aug 3;21:151. doi: 10.1186/s12862-021-01883-w (PMC8330473; doi:10.1186/s12862-021-01883-w)
Supplement: Supplementary file 3 — Additional file 3. Match between natural areas and target paint colours in field trials. [file 12862_2021_1883_MOESM3_ESM.pdf]

**<u>Additional file 3:</u> Match between natural areas and target paint colours in field trials**

*Methods*

To check how well models matched the specific background areas they were chosen to represent during the experiment, we compared the colours of the painted models to the whole visual scenes and specific representative areas of bracken, grass, bramble and leaf litter in photographs of the target locations used in the field trials, following methods used for identifying the best paint colours in the first place. Representative areas were selected from photographs of the pink hare model *in situ*, facing right, taken from 10m away with the same photography equipment, and a 70mm zoom. One woodland image (position 6 along the transect) did not feature any sufficiently large patches of bramble or leaf litter, so specialist areas were not selected from this image, yielding total samples of $N_{bracken} = N_{grass} = 20$, $N_{bramble} = N_{leaf\ litter} = 19$, $N_{whole\ scene} = 40$ to compare to the model colours.

The variation in colour difference between paints and natural areas was tested with a series of mixed effects models, run using the packages 'lme4' [1] and 'lmerTest' [2]. An initial model was run with $\Delta E$ values as the response variable, natural area and paint name as fixed effects, and unique field positions as a random effect to account for multiple measurements of each area. Evidence of a significant interaction between paint colour and natural area (linear mixed effects model, $\chi^2 = 834.45$, $df = 36$, $p < 0.001$) led to two series of mixed effects models. The first was designed to test the differences between all paint colours in matching each area; data were filtered by natural area, and paint colour was the only fixed effect, with position as a random effect. A second series of models tested how similar each paint colour was to all natural areas; data were filtered by paint colour, and each model included only natural area as a fixed effect, with the same random effect. Likelihood ratio tests were used to assess the significance of the fixed effects, and Tukey's post-hoc tests relative to a reference group were carried out using the glht function in the package 'multcomp' [3]. For the first series of models, the reference paint for each natural area was one of the two paints selected to represent that area, chosen as a reference to be the one with the highest colour difference of the two, so that any significant increase in contrast for other paints compared to the reference would also indicate a worse match than the other representative paint. In the second series of models, the reference level corresponded to the natural area each paint type should be matching. Diagnostic plots were used to verify the assumptions of mixed effects models, and data for some models were square-root transformed to fit these assumptions.

*Results*

Colour difference (in ΔE) between paint colours and natural areas varied significantly between natural areas, for every paint type (see Supplementary Table 2). Post-hoc tests, relative to the area each paint was chosen to match, confirm that no paints were a better match to a different area than to the one they were meant to represent (Supplementary Table 3). Paints all had significantly higher colour difference values to other areas relative to the reference, with the exception of both paints matching bracken, which were similarly well matched to the whole image selections, and one generalist paint ('Florentine Dream'), which matched bramble selections equally well. Flipping the analysis to test how well each natural area was matched by the different paints also found a significant difference between paint colours for all areas (Supplementary Table 4), and confirmed no paints were a better match than those chosen to represent each area (Supplementary Table 5). All paints were more different from the areas they were not chosen to match than their representative paints, with exceptions only for the bracken area (similarly well matched by paints matching leaf litter and the whole image selections), and bramble area (equally well matched by one paint matching grass selections ['Pressed Olives'] and one paint matching whole image selections ['Wagon Train']). Therefore, in our experiment, all specialist targets were well matched to a specific area, but only the grass and leaf litter models fulfilled a stricter criterion for a specialist strategy, better matching their specialist area than any other targets do, including generalists.

Supplementary Table 2: Tests of significance of the effect of natural area in all paint colour models. Distance in CIELab (ΔE) was square root-transformed in models for 'Char Latte', 'Toffee Coffee', 'Chartreuse', 'Herb Garland'.

| **Paint colour** | $\chi^2$ | **df** | **p** |
|---|---|---|---|
| 'Aged Cognac' | 64.297 | 4 | < 0.001 |
| 'Village Pub' | 67.783 | 4 | < 0.001 |
| 'Char Latte' | 73.243 | 4 | < 0.001 |
| 'Toffee Coffee' | 118.48 | 4 | < 0.001 |
| 'Chartreuse' | 92.78 | 4 | < 0.001 |
| 'Pressed Olives' | 98.217 | 4 | < 0.001 |
| 'Herb Garland' | 167.97 | 4 | < 0.001 |
| 'Leafy Greens' | 168.92 | 4 | < 0.001 |
| 'Florentine Dream' | 112.82 | 4 | < 0.001 |
| 'Wagon Train' | 79.716 | 4 | < 0.001 |

Supplementary Table 3: Results of post-hoc tests for models testing differences in colour difference (ΔE) between target paint colours and selections from all possible natural areas. For all paints, colour difference was lowest for the area they were chosen to represent, and most other areas were significantly more contrasting; rare cases when other areas were similarly well-matched are highlighted in italic.

| **Paint** | **Natural area** | **Estimate** | **Std. error** | **z value** | **p value** |
|---|---|---|---|---|---|
| 'Aged Cognac' | Bracken (reference) | 11.444 | 1.207 | 9.485 | < 0.001 |
| | Bramble | 5.551 | 1.679 | 3.306 | 0.00414 |
| | Grass | 12.457 | 1.438 | 8.660 | < 0.001 |
| | Leaf litter | 5.517 | 1.679 | 3.286 | 0.00464 |
| | *Whole image* | *2.045* | *1.312* | *1.558* | *0.347* |
| 'Village Pub' | Bracken (reference) | 11.296 | 1.186 | 9.523 | < 0.001 |
| | Bramble | 6.536 | 1.654 | 3.951 | <0.001 |
| | Grass | 13.170 | 1.424 | 9.251 | < 0.001 |
| | Leaf litter | 4.998 | 1.654 | 3.021 | 0.0103 |
| | *Whole image* | *2.863* | *1.296* | *2.209* | *0.0957* |
| 'Char Latte' | Leaf litter (reference) | 3.168 | 0.145 | 21.905 | < 0.001 |
| | Bracken | 0.493 | 0.199 | 2.481 | 0.0486 |
| | Bramble | 1.609 | 0.179 | 8.983 | < 0.001 |
| | Grass | 1.299 | 0.199 | 6.542 | < 0.001 |
| | Whole image | 1.204 | 0.161 | 7.485 | < 0.001 |
| 'Toffee Coffee' | Leaf litter (reference) | 2.893 | 0.141 | 20.519 | < 0.001 |
| | Bracken | 0.817 | 0.195 | 4.179 | < 0.001 |
| | Bramble | 2.173 | 0.182 | 11.912 | < 0.001 |
| | Grass | 2.108 | 0.195 | 10.790 | < 0.001 |
| | Whole image | 1.772 | 0.162 | 10.970 | < 0.001 |
| 'Chartreuse' | Grass (reference) | 2.810 | 0.138 | 20.422 | < 0.001 |
| | Bracken | 1.733 | 0.166 | 10.432 | < 0.001 |
| | Bramble | 1.294 | 0.192 | 6.728 | < 0.001 |
| | Leaf litter | 1.946 | 0.192 | 10.123 | < 0.001 |
| | Whole image | 1.142 | 0.151 | 7.564 | < 0.001 |

| | | | | | |
|---|---|---|---|---|---|
| 'Pressed Olives' | Grass (reference) | 6.727 | 0.963 | 6.984 | < 0.001 |
| | Bracken | 10.839 | 1.348 | 8.038 | < 0.001 |
| | Bramble | 4.424 | 1.380 | 3.206 | 0.00569 |
| | Leaf litter | 14.287 | 1.380 | 10.354 | < 0.001 |
| | Whole image | 3.868 | 1.172 | 3.301 | 0.00360 |
| 'Herb Garland' | Bramble (reference) | 2.896 | 0.107 | 27.248 | < 0.001 |
| | Bracken | 1.830 | 0.142 | 12.867 | < 0.001 |
| | Grass | 1.151 | 0.142 | 8.093 | < 0.001 |
| | Leaf litter | 2.308 | 0.122 | 18.947 | < 0.001 |
| | Whole image | 0.636 | 0.112 | 5.692 | < 0.001 |
| 'Leafy Greens' | Bramble (reference) | 11.310 | 0.887 | 12.753 | < 0.001 |
| | Bracken | 13.771 | 1.165 | 11.824 | < 0.001 |
| | Grass | 9.130 | 1.165 | 7.839 | < 0.001 |
| | Leaf litter | 18.910 | 0.975 | 19.401 | < 0.001 |
| | Whole image | 4.303 | 0.904 | 4.762 | < 0.001 |
| 'Florentine Dream' | Whole image (reference) | 5.889 | 0.674 | 8.738 | < 0.001 |
| | Bracken | 6.893 | 0.978 | 7.046 | < 0.001 |
| | *Bramble* | *2.316* | *0.999* | *2.319* | *0.0895* |
| | Grass | 8.907 | 0.978 | 9.106 | < 0.001 |
| | Leaf litter | 12.687 | 0.999 | 12.700 | < 0.001 |
| 'Wagon Train' | Whole image (reference) | 7.471 | 0.772 | 9.679 | < 0.001 |
| | Bracken | 3.295 | 1.117 | 2.949 | 0.0150 |
| | Bramble | 3.571 | 1.141 | 3.129 | 0.00835 |
| | Grass | 10.057 | 1.117 | 9.001 | < 0.001 |
| | Leaf litter | 9.475 | 1.141 | 8.302 | < 0.001 |

Supplementary Table 4: Tests of significance of the effect of paint colour in all natural area models.

| **Natural area** | $\chi^2$ | **df** | **p** |
|---|---|---|---|
| Bracken | 104.66 | 9 | < 0.001 |
| Bramble | 222.61 | 9 | < 0.001 |
| Grass | 230.53 | 9 | < 0.001 |
| Leaf litter | 220.19 | 9 | < 0.001 |
| Whole image | 368.28 | 9 | < 0.001 |

Supplementary Table 5: Results of post-hoc tests for models testing differences in colour difference (ΔE) between natural areas and all target paints. For all areas, colour difference was lowest for the paints chosen to represent each area, and most other paints were significantly more contrasting; rare cases when other paints were similarly well-matched are highlighted in italic.

| **Natural area** | **Colour** | **Estimate** | **Std. error** | **z value** | **p value** |
|---|---|---|---|---|---|
| Bracken | 'Aged Cognac' (ref) | 11.722 | 1.326 | 8.843 | < 0.001 |
| | 'Village Pub' | -0.128 | 1.781 | -0.072 | 1.000 |
| | *'Char Latte'* | *2.982* | *1.781* | *1.674* | *0.449* |
| | *'Toffee Coffee'* | *3.281* | *1.781* | *1.842* | *0.340* |
| | 'Chartreuse' | 9.242 | 1.781 | 5.189 | < 0.001 |
| | 'Pressed Olives' | 5.837 | 1.781 | 3.277 | 0.00866 |
| | 'Herb Garland' | 10.344 | 1.781 | 5.807 | < 0.001 |
| | 'Leafy Greens' | 12.951 | 1.781 | 7.271 | < 0.001 |
| | *'Florentine Dream'* | *1.110* | *1.781* | *0.623* | *0.996* |
| | *'Wagon Train'* | *-0.708* | *1.781* | *-0.398* | *1.000* |
| Bramble | 'Leafy Greens' (ref) | 11.761 | 0.906 | 12.984 | < 0.001 |
| | 'Herb Garland' | -2.762 | 1.204 | -2.294 | 0.137 |
| | 'Aged Cognac' | 4.942 | 1.204 | 4.104 | < 0.001 |
| | 'Village Pub' | 5.757 | 1.204 | 4.781 | < 0.001 |
| | 'Char Latte' | 10.948 | 1.204 | 9.093 | < 0.001 |
| | 'Toffee Coffee' | 13.783 | 1.204 | 11.448 | < 0.001 |
| | 'Chartreuse' | 5.537 | 1.204 | 4.599 | < 0.001 |
| | *'Pressed Olives'* | *-0.600* | *1.204* | *-0.499* | *0.999* |
| | 'Florentine Dream' | -3.566 | 1.204 | -2.962 | 0.0236 |

| | | | | | |
|---|---|---|---|---|---|
| | *'Wagon Train'* | *-0.966* | *1.204* | *-0.803* | *0.976* |
| Grass | 'Chartreuse' (ref) | 8.523 | 1.193 | 7.146 | < 0.001 |
| | 'Pressed Olives' | -1.803 | 1.305 | -1.381 | 0.684 |
| | 'Herb Garland' | 7.851 | 1.305 | 6.015 | < 0.001 |
| | 'Leafy Greens' | 11.509 | 1.305 | 8.818 | < 0.001 |
| | 'Aged Cognac' | 15.656 | 1.305 | 11.994 | < 0.001 |
| | 'Village Pub' | 16.241 | 1.305 | 12.442 | < 0.001 |
| | 'Char Latte' | 11.925 | 1.305 | 9.136 | < 0.001 |
| | 'Toffee Coffee' | 16.891 | 1.305 | 12.491 | < 0.001 |
| | 'Florentine Dream' | 6.323 | 1.305 | 4.844 | < 0.001 |
| | 'Wagon Train' | 9.253 | 1.305 | 7.089 | < 0.001 |
| Leaf litter | 'Char Latte' (ref) | 10.272 | 1.196 | 8.588 | < 0.001 |
| | 'Toffee Coffee' | -1.544 | 1.428 | -1.081 | 0.881 |
| | 'Chartreuse' | 12.464 | 1.428 | 8.726 | < 0.001 |
| | 'Pressed Olives' | 10.752 | 1.428 | 7.527 | < 0.001 |
| | 'Herb Garland' | 17.547 | 1.428 | 12.285 | < 0.001 |
| | 'Leafy Greens' | 20.398 | 1.428 | 14.281 | < 0.001 |
| | 'Aged Cognac' | 6.396 | 1.428 | 4.478 | < 0.001 |
| | 'Village Pub' | 5.707 | 1.428 | 3.996 | < 0.001 |
| | 'Florentine Dream' | 8.294 | 1.428 | 5.806 | < 0.001 |
| | 'Wagon Train' | 6.426 | 1.428 | 4.499 | < 0.001 |
| Whole image | 'Wagon Train' (ref) | 7.471 | 0.610 | 12.240 | < 0.001 |
| | 'Florentine Dream' | -1.582 | 0.863 | -1.833 | 0.343 |
| | 'Char Latte' | 11.869 | 0.863 | 13.752 | < 0.001 |
| | 'Toffee Coffee' | 14.463 | 0.863 | 16.756 | < 0.001 |
| | 'Chartreuse' | 8.554 | 0.863 | 9.911 | < 0.001 |
| | 'Pressed Olives' | 3.124 | 0.863 | 3.619 | 0.00258 |
| | 'Herb Garland' | 5.163 | 0.863 | 5.982 | < 0.001 |
| | 'Leafy Greens' | 8.142 | 0.863 | 9.433 | < 0.001 |
| | 'Aged Cognac' | 6.018 | 0.863 | 6.972 | < 0.001 |
| | 'Village Pub' | 6.688 | 0.863 | 7.748 | < 0.001 |

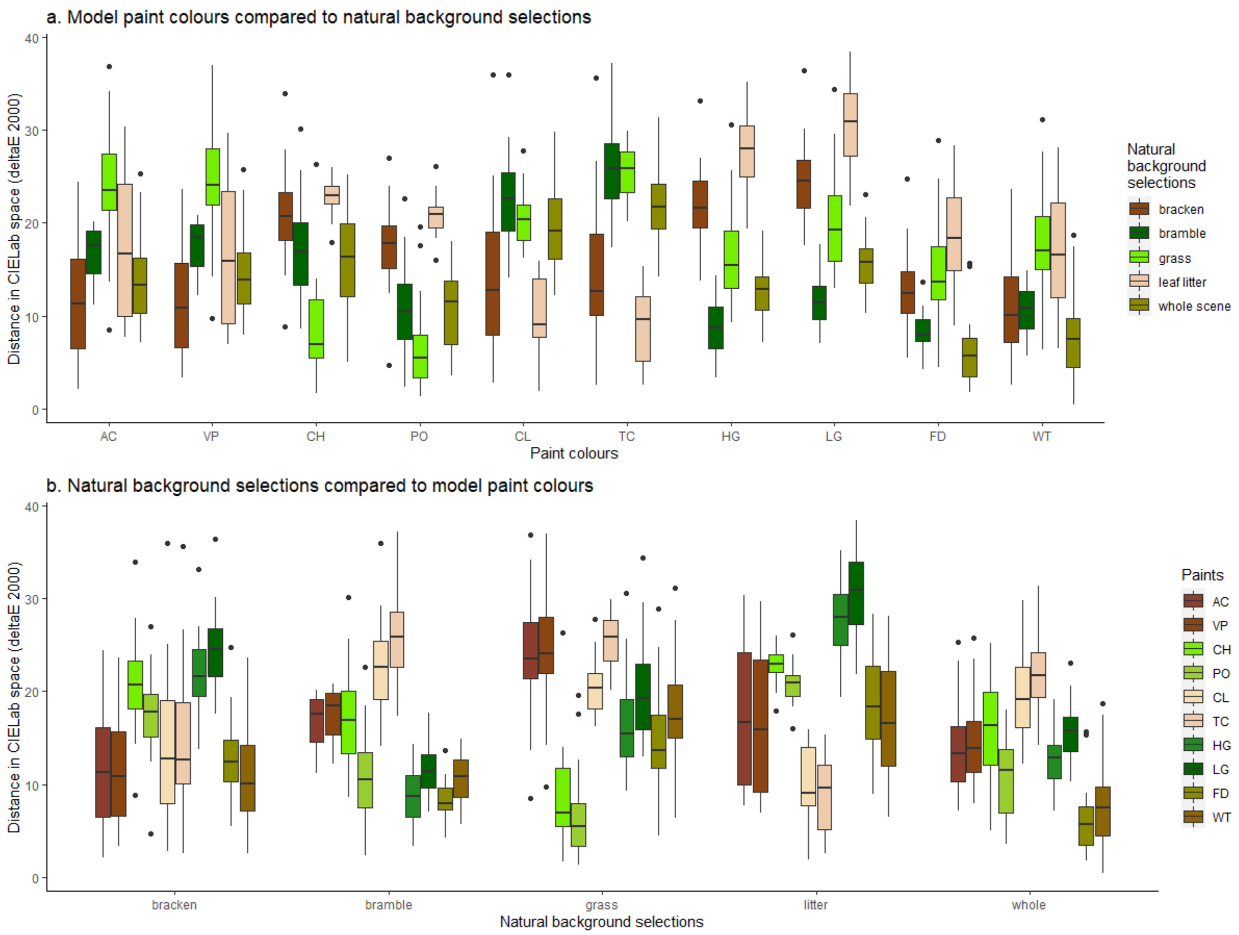


Supplementary Figure 4: ΔE values between model paints and the natural backgrounds they were chosen to represent, showing how well each paint matched the different areas (a) and how similar each area was to the different paints selected (b).

## References


1. Bates D, Maechler M, Bolker B, Walker S. Fitting linear mixed-effects models using lme4. *J Stat Softw.* 2015;67:1-48.

2. Kuznetsova A, Brockhoff PB, Christensen RHB. lmerTest Package: Tests in linear mixed effects models. *J Stat Softw.* 2017;82:1-26.

3. Hothorn T, Bretz F,Westfall P. Simultaneous inference in general parametric models. *Biom J.* 2008;50:346-363.
