# Supplementary material for: Generalist camouflage can be more successful than microhabitat specialisation in natural environments
Source: BMC Ecol Evol. 2021 Aug 3;21:151. doi: 10.1186/s12862-021-01883-w (PMC8330473; doi:10.1186/s12862-021-01883-w)
Supplement: Supplementary file 4 — Additional file 4. Paint colours used in field trials. [file 12862_2021_1883_MOESM4_ESM.pdf]

Additional file 4: Paint colours used in field trials.

Supplementary Table 6: CIELab values of target natural colours and chosen paint colours (as sample cards and actually painted on models), and quantification of colour differences between them.

**Strategy**	**Paint colour name**	**Median colour of the target natural areas**	**Colour of chosen paint sample card**	**Distance between target median & paint card colour (CIEDE2000)**	**Colour of chosen paint**	**Distance between target median & paint (CIEDE2000)**
Specialist - grass	"Chartreuse" R228B	L = 48.889<br>a = -12.702<br>b = 36.514	L = 55.020<br>a = -10.127<br>b = 39.169	6.472	L = 61.043<br>a = -10.820<br>b = 44.831	12.109
	"Pressed Olives" R239A	L = 48.889<br>a = -12.702<br>b = 36.514	L = 51.396<br>a = -9.328<br>b = 34.562	3.283	L = 55.095<br>a = -8.731<br>b = 35.166	6.634
Specialist - bracken	"Aged Cognac" R101F	L = 42.232<br>a = 9.945<br>b = 23.479	L = 37.667<br>a = 9.962<br>b = 17.182	5.479	L = 42.182<br>a = 10.824<br>b = 21.923	1.377
	"Village Pub" R100F	L = 42.232<br>a = 9.945<br>b = 23.479	L = 38.517<br>a = 12.058<br>b = 18.059	5.454	L = 42.778<br>a = 12.350<br>b = 22.722	2.289
Specialist - leaf litter	"Char Latte" R106D	L = 58.350<br>a = 12.916<br>b = 24.107	L = 59.724<br>a = 9.981<br>b = 25.537	3.063	L = 65.542<br>a = 10.627<br>b = 31.536	7.801
	"Toffee Coffee" R101D	L = 58.350<br>a = 12.916<br>b = 24.107	L = 58.880<br>a = 15.738<br>b = 21.839	3.078	L = 64.762<br>a = 17.307<br>b = 28.286	6.222
Specialist - bramble	"Herb Garland" R262A	L = 41.079<br>a = -6.603<br>b = 21.972	L = 42.525<br>a = -8.520<br>b = 16.813	4.018	L = 44.046<br>a = -10.180<br>b = 16.866	5.548
	"Leafy Greens" R261A	L = 41.079<br>a = -6.603<br>b = 21.972	L = 40.511<br>a = -7.802<br>b = 12.980	5.740	L = 41.529<br>a = -10.354<br>b = 11.865	7.684
Generalist	"Florentine Dream" R108F	L = 44.777<br>a = 1.796<br>b = 22.399	L = 45.21 3<br>a = 1.196<br>b = 23.949	3.160	L = 45.389<br>a = 0.316<br>b = 26.649	2.594
	"Wagon Train" R106F	L = 44.777<br>a = 1.796<br>b = 22.399	L = 42.830<br>a = 4.765<br>b = 22.882	3.844	L = 44.624<br>a = 4.335<br>b = 24.871	2.554
