# Supplementary material for: Generalist camouflage can be more successful than microhabitat specialisation in natural environments
Source: BMC Ecol Evol. 2021 Aug 3;21:151. doi: 10.1186/s12862-021-01883-w (PMC8330473; doi:10.1186/s12862-021-01883-w)
Supplement: Supplementary file 5 — Additional file 5. Field trial analyses, repeated with restricted datasets. [file 12862_2021_1883_MOESM5_ESM.pdf]

Additional file 5: **Field trial analyses, repeated with restricted datasets**

- Survival of generalist versus specialist targets in the field

Trends seen with the full dataset were borne out even when results were restricted to participants who completed trials in both field sites (N = 15, coxme, effect of strategy: χ^2_1 = 24.201, HR = 0.573, CI = 0.455-0.723, z = -4.7, p < 0.001), and when excluding the single colour-blind participant (N = 14, coxme, effect of strategy: χ^2_1 = 24.086, HR = 0.560, CI = 0.440-0.714, z = -4.69, p < 0.001).

- Survival of targets in the field, according to target colour

Supplementary Table 7: Model simplification tables for survival models testing the effects of weather, habitat and target colour on survival probability.

a. Dataset restricted to 15 participants, who completed both trials

**Model**	**Factor**	χ^2	**df**	**p**
**Across both habitats**	Order	0.573	1	0.449
	Weather:Habitat	0.851	1	0.356
	Weather:Colour	13.325	9	0.148
	Weather	0.185	1	0.667
	Colour:Habitat	19.897	9	0.0186
**Farm only**	Order	0.0085	1	0.926
	Colour:Weather	4.695	9	0.860
	Weather	0.924	1	0.336
	Colour	42.727	9	$2.419*10^{-6}$ †
**Wood only**	Order	1.136	1	0.287
	Colour:Weather	10.976	9	0.277
	Weather	0.437	1	0.509
	Colour	63.436	9	$2.904*10^{-10}$ ‡

†: Key comparisons in the farm - All specialist colours survive worse than or similarly to generalists.

‡: Key comparisons in the wood - All specialist colours survive worse than or similarly to generalist 1; all survive worse than or similarly to generalist 2, except generalist 1 (HR = 0.444, CI = 0.242-0.814) & "Pressed Olives" (grass specialist, HR = 0.366, CI = 0.196-0.685).

b. Dataset restricted to 14 participants, excluding a colour-blind participant

Model	Factor	χ^2	df	p
**Across both habitats**	Order	0.951	1	0.329
	Weather:Habitat	0.546	1	0.460
	Weather:Colour	16.045	9	0.0660
	Colour:Habitat	14.046	9	0.121
	Weather	0.011	1	0.916
	Habitat	1.127	1	0.286
	Colour	89.514	9	$1.998*10^{-15}$ *

*: Key comparisons - All specialist colours survive worse than or similarly to generalist 1; all survive worse than or similarly to generalist 2, except generalist 1 (HR = 0.483, CI = 0.313-0.745) & "Pressed Olives" (grass specialist, HR = 0.555, CI = 0.361-0.853).

- Survival of targets in the field, based on colour differences between targets and backgrounds

Supplementary Table 8: Effects of colour difference in ΔE (CIEDE2000) between targets and backgrounds on detection risk in survival models, for restricted datasets. Models are ranked by increasing AIC, including the model testing the effect of strategy (generalist/specialist) for comparison. Hazard ratios (HR) >1 indicate that increasing difference between models and backgrounds increases detection risk. The HR for the Strategy model corresponds to a lower risk for generalists relative to specialists.

a. Dataset restricted to 15 participants, who completed both trials

**Explanatory variable**								
**Colour difference metric**	**Area**	**Distance from model**	**z**	**HR**	**CI**	χ^2_1	**p**	**AIC**
ΔE (CIEDE2000)	Near zone	10m	7.68	1.065	1.048 -1.083	60.437	<0.001	5767.151
ΔE (CIEDE2000)	Near zone	30m	6.68	1.054	1.038 -1.071	45.24	<0.001	5782.068
ΔE (CIEDE2000)	Whole image	10m	6.38	1.052	1.036 - 1.068	41.226	<0.001	5787.455
ΔE (CIEDE2000)	Whole image	30m	5.43	1.045	1.029 - 1.062	29.791	<0.001	5798.761
Strategy	-	-	-4.7	0.573	0.455 - 0.723	24.201	<0.001	5803.967
Null	-	-	-	-	-	-	-	5828.760

b. Dataset restricted to 14 participants, excluding the colour-blind participant.

**Explanatory variable**								
**Colour difference metric**	**Area**	**Distance from model**	**z**	**HR**	**CI**	χ^2_1	**p**	**AIC**
ΔE (CIEDE2000)	Near zone	10m	7.56	1.066	1.048 -1.084	58.383	<0.001	5318.495
ΔE (CIEDE2000)	Near zone	30m	6.3	1.053	1.036 -1.070	40.321	<0.001	5337.210
ΔE (CIEDE2000)	Whole image	10m	6.23	1.052	1.036 - 1.069	39.326	<0.001	5339.194
ΔE (CIEDE2000)	Whole image	30m	5.47	1.047	1.030 - 1.065	30.169	<0.001	5348.015
Strategy	-	-	-4.69	0.560	0.440 -0.714	24.086	<0.001	5353.918
Null	-	-	-	-	-	-	-	5379.727

- Survival of microhabitat specialist targets in the field, based on the proportion of areas occupied by different patch types

As with the full dataset, when data are restricted to participants who completed trials in both field sites (N=15), the proportion of the area in the whole visual scene occupied by the same microhabitat as represented by the target had no effect on detection risk (coxme, $\chi^2_1 = 0.0208$, HR = 0.962, CI = 0.570-1.622, z = -0.15, p = 0.885), but there was a significant effect when this proportion was calculated for a restricted near zone around the target, (coxme, $\chi^2_1 =$ 4.343, HR = 0.597, CI = 0.365-0.976, z = -2.06, p = 0.0372). However, neither variable is significant when the colour-blind participant is further excluded (N=14, coxme, $\chi^2_1 = 0.215$, HR = 1.136, CI = 0.664-1.944, z = 0.47, p = 0.643 & $\chi^2_1 = 2.595$, HR = 0.661, CI = 0.398-1.099, z = -1.59, p = 0.107 for proportion area in the whole image and near zone resp.).
