# Supplementary material for: Generalist camouflage can be more successful than microhabitat specialisation in natural environments
Source: BMC Ecol Evol. 2021 Aug 3;21:151. doi: 10.1186/s12862-021-01883-w (PMC8330473; doi:10.1186/s12862-021-01883-w)
Supplement: Supplementary file 6 — Additional file 6. Methods for analyses of target camouflage. [file 12862_2021_1883_MOESM6_ESM.pdf]

Additional file 6: Methods for analyses of target camouflage

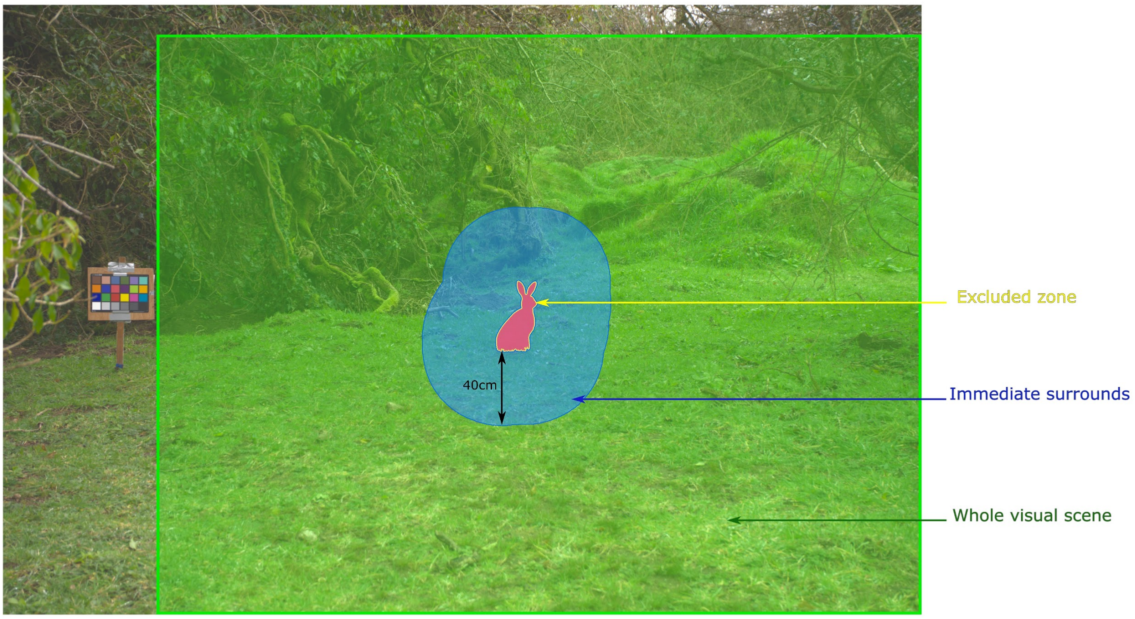

Supplementary Figure 5: Areas of interest for analysis of natural background images. The pink model hare allowed us to pinpoint the exact location of the targets in that position. We analysed coloration in the immediate surrounds of the model hare (blue zone, a band 40cm wide, corresponding to the height of a model hare) and the whole visual scene around the model, excluding the skyline and colour standards (green zone). The exclusion zone around the model is shown in yellow.
